# Blind spots in medical education: how can we envision new possibilities?

**DOI:** 10.1007/s40037-022-00730-y

**Published:** 2022-11-22

**Authors:** Sean Tackett, Yvonne Steinert, Cynthia R. Whitehead, Darcy A. Reed, Scott M. Wright

**Affiliations:** 1 Division of General Internal Medicine, Johns Hopkins Bayview Medical Center, Baltimore, MD USA; 2 Faculty of Medicine and Health Sciences, McGill University, Montreal, Canada; 3 Wilson Centre for Research in Education, University of Toronto, Toronto, Ontario Canada; 4 Department of Family and Community Medicine, Faculty of Medicine, University of Toronto, Toronto, Ontario Canada; 5 Division of Community Internal Medicine, Mayo Clinic, Rochester, MN USA; 6 College of Medicine and Science, Mayo Clinic, Rochester, MN USA

**Keywords:** Biases, Blind spots, Medical education reform, Innovation

## Abstract

As human beings, we all have blind spots. Most obvious are our visual blind spots, such as where the optic nerve meets the retina and our inability to see behind us. It can be more difficult to acknowledge our other types of blind spots, like unexamined beliefs, assumptions, or biases. While each individual has blind spots, groups can share blind spots that limit change and innovation or even systematically disadvantage certain other groups. In this article, we provide a definition of blind spots in medical education, and offer examples, including unfamiliarity with the evidence and theory informing medical education, lack of evidence supporting well-accepted and influential practices, significant absences in our scholarly literature, and the failure to engage patients in curriculum development and reform. We argue that actively helping each other see blind spots may allow us to avoid pitfalls and take advantage of new opportunities for advancing medical education scholarship and practice. When we expand our collective field of vision, we can also envision more “adjacent possibilities,” future states near enough to be considered but not so distant as to be unimaginable. For medical education to attend to its blind spots, there needs to be increased participation among all stakeholders and a commitment to acknowledging blind spots even when that may cause discomfort. Ultimately, the better we can see blind spots and imagine new possibilities, the more we will be able to adapt, innovate, and reform medical education to prepare and sustain a physician workforce that serves society’s needs.

Long ago, Aristotle ranked sight as first among the five senses. Vision has since been the subject of popular aphorisms in the English language, from “seeing is believing” to “I can’t believe my eyes,” and the subject of a disproportionate amount of scientific research [1].

While vision may be the best understood and most valued sense, it remains flawed. Humans have physiologic blind spots in each eye where the optic nerve meets the retina. One can go an entire lifetime without being aware of this blind spot, as we have evolved for it to be imperceptible. Our field of vision spans only the 210 degrees in front of us, leaving us unable to see what’s behind us—unless we turn our heads. What we perceive is further limited by our attention. We have an overwhelming amount of visual information before us; yet we can only truly “see” one thing at a time (consider ambiguous images where you must switch between two different image interpretations). We can notice more of what is in our field of view, but it takes time and effort to do so. Finally, optical illusions demonstrate that our vision can be distorted to the point that we see things that are not there at all. We generally rely on others to help us overcome these distortions and become aware of what the Johari window referred to as the “blind area,” something that is unknown to oneself but known by others [2]. However, we can be collectively blind as well—particularly if we are all looking in the same direction and ignoring what is behind us, or not exploring what may be in the dark.

The term “blind spot” is now also commonly used to refer to the unexamined beliefs, assumptions, or biases of an individual or group. Titles from popular books and other media have referred to blind spots that cause individuals to make poor decisions at work or in business [3], or that represent a misunderstanding of historical events, propagated by sociocultural structures and inequities [4]. Similar to visual blind spots, without changing direction or seeking help from others, one can go an entire lifetime without being aware of personal biases and assumptions. This can perpetuate disparities, especially when such blind spots are unacknowledged by those with power and privilege.

If we accept that every human being has a variety of blind spots, it follows that those involved in medical education scholarship and practice have them too. In this article, we suggest that actively looking for our blind spots can provide a useful approach to advancing meaningful reform in medical education.

## Problems in medical education from inattention to blind spots and the need for a new approach

Medical education systems tend to focus on developing an individual’s clinical expertise to prepare them for independent practice, rather than producing and sustaining a clinician workforce that is poised to meet society’s needs. This occurs across all stages of the medical education continuum. For example, medical school admissions processes are designed to ensure a student’s successful progression through the curriculum, but the demographics of those selected to become physicians are typically discordant with those of the broader population from which they are drawn [5]. During medical school, students build a foundation of clinical knowledge and skills, yet their well-being begins a decline that persists into postgraduate training and throughout their professional careers; this is known to adversely impact interactions with patients and colleagues [6]. After physicians complete their structured training programs, clinical outcomes of patients under their care are inversely correlated with the physician’s years in practice; this may point to suboptimal continuing medical education systems [7]. These problems are well-recognized, yet persistent. An analysis of calls for reform in medical education found recurrent themes over a 100-year period [8].

Factors preventing reform are sundry. Some factors directly affect educators. For example, promotions criteria for the advancement of faculty members through academic ranks frequently assign premium value to grants and peer-reviewed publications. This arrangement affords significant influence to external funders and biomedical journals in setting the research agenda and promoting scholarship that is aligned with their own priorities. Faculty members are often compelled to follow established priorities, responding to requests for proposals, and conducting relatively “safe” research that is most likely to be understood by reviewers and editors. Being bold and proposing original ideas may even slow one’s path to academic advancement.

Medical education scholarship and practice is often conducted in institutions and clinical settings that seek to align with health system needs and financial realities. Clinical programs that expand revenue and other activities that enhance institutional reputation are often prioritized [9]. Without an obvious return on investment, education rarely receives the resources needed to innovate, evaluate, and improve to keep pace with changes in clinical practice.

Medical education and healthcare more generally take place within societies. The historical experiences and sociocultural statuses of individuals and groups in these societies inherently confer privilege to some, disadvantage others, and create and reproduce hierarchies. Evidence shows that globally, systemic and structural disparities have been worsening [10].

In short, medical education needs disruptive thinking and action if it is to better serve society’s health needs, as existing structures and those who have power within them may constrain the identification and pursuit of new and promising ideas from across the community. By virtue of the fact that a limited number of individuals are in a position to shape the overall direction of medical education, we all become subject to their blind spots.

## What are blind spots in medical education?

Blind spots in medical education can be thought of as issues or ideas that are not seen or not receiving enough attention; greater acknowledgement and responsiveness to blind spots may result in transformative change.

Individuals can have blind spots. Evidence suggests that many are blind to, or unfamiliar with, the research being accrued in medical education [11]. This lack of awareness can lead to the perpetuation of common “myths” [12] that may prevent the adoption of effective educational practices. Someone’s vision may also be obscured when they are too close to something (e.g., their own sleep deprivation or compromised well-being); stepping back may be needed to refocus and obtain perspective. Trying to see one’s implicit biases, which can profoundly influence how they see the world, make decisions, and render judgments, often requires effort and may be accompanied by discomfort when seeking to reduce their negative influences [13].

Groups can share blind spots. In medicine, we are prone to assume that common practices are evidence-based. However accreditation requirements for medical schools, which broadly influence medical education systems, have developed, in part, based on tradition and opinion with limited examination by systematic inquiry [14]. Technologic innovations are another example, where some, like point of care ultrasound, can propagate based on unfounded enthusiasm despite a lack of evidence [15]. Foundational concepts that we might assume are shared, such as what it means to be a good doctor, can change over time [16] and vary widely depending on contexts and perspectives [17]. Even well-intentioned efforts to reduce global disparities by increasing educational capacity in lower resourced settings, can create cultural hegemonies if educators from higher resourced settings are inattentive to their blind spots [18].

While many blind spots among individuals and groups can be due to a failure to consider something that has been plainly in the field of view (e.g., the literature on professional identity formation has thus far been blind to important racial and minority issues [19]), blind spots may also be created if we make something disappear [20]. For example, the notion of compassionate care has gradually been removed from the standards that define expectations for educational programs [21, 22].

Some of medical education’s most persistent problems are due to blind spots created by insufficient attention over time. While we may see some problems clearly for a moment, distractions or competing priorities can cause us to turn our attention away before they are fully addressed. For example, many have called for greater patient involvement in medical education [23], including in curriculum design [24, 25], and useful guides for involving patients in medical education have been published [26]. Nonetheless, there are few published examples of patients engaged in substantive ways in curriculum development. Indeed, the reasons why medical education fails to involve patients may be complex, and entangled in physicians’ social position, in relation to patients. Evidence suggests that physicians may be disinclined to truly empower patients, because this may entail giving up some power of their own [27].

## Why we should look for blind spots in medical education

Evidence from the neurosciences indicates that our frames of reference determine what we are capable of seeing and thinking and subsequently the new ideas that can be pursued [28]. Political and social sciences describe a similar phenomenon, as powerful actors compete to frame problems that make their own interests and priorities more visible and influential [29]. Moreover, human beings have shared thought patterns, resulting from millions of years of evolution, that may need to be overcome intentionally in many of today’s societies if we are to serve a greater good—such as improving the health and life experiences of all individuals [30]. Metaphors can align groups and cultures behind common ways of thinking and engender new cultural norms [31]. Collectively and systematically looking for blind spots offers a new metaphor and a framing that centers on the innate flaws that each of us has; thinking in this way may make it easier to acknowledge a variety of blind spots that require more attention. This could further help us recognize complexity, achieve inclusion from all stakeholders, level hierarchies, and bring coherence to disparate conversations with respect to reform in medical education.

One contemporary example of where a frame shift that incorporates the idea of blind spots could be helpful is the problem of health inequities. Calls to reduce inequities often have supporters arguing for one side against another, leading to a binary bias, where a complex continuum is simplified into two categories [32]. Advocates may further be positioned as competing with one another over which inequity is most important, rather than looking for the merits of all points of view so as to resolve tensions and realize shared outcomes [33]. Unquestionably, all have a role to play in reducing inequities. Reforming longstanding structures that privilege some while disadvantaging others will not be easy or happen quickly. Yet, we suggest that if we reframe the problem of inequities as the result of blind spots that each of us inevitably possesses, it may become less threatening to engage in challenging conversations. Our starting point would become trying to see our own blind spots by inviting the perspectives of those who think differently rather than defending our stance or putting others on the defensive. “Complexifying” in this way could increase collective learning and facilitate cooperation [34]. An awareness that blind spots are pervasive may decrease confrontation and blaming, and instead promote honesty, transparency, and humility.

Not only might it be useful to reframe certain problems as being the result of blind spots, as medical education scholars, we are part of a tradition where we should be the first to examine our own blind spots. For example, definitions of scholarly inquiry require critical reflection on one’s work to point out its limitations. Currently, however, drawing attention to potential weaknesses is disincentivized as the pressure to publish remains, and there is growing acceptance of promoting one’s “brand” [35]. The peer review process is intended to illuminate blind spots not seen by those closest to a project, although reviewers themselves are likely to have blind spots. Peer reviewers placed into a position of authority, for example, may have difficulty acknowledging their lack of familiarity with a methodology or subject matter. Placing appropriate emphasis on the blind spots that inevitably limit our scholarship will strengthen it—and the trust placed in it by others.

The timing may be right to focus on medical education’s blind spots. The COVID-19 pandemic unleashed disruptive forces that can create the type of unfreezing that facilitates meaningful change [36]. Leading medical education journals have been promoting discussions about myths [12], seeking to accommodate diverse perspectives [37], and questioning the habit of looking for simple solutions that are intended to address complex problems [38]. Professional organizations are complexifying by supporting systems-thinking to confront healthcare challenges [39] and expressing urgency to enhance collaboration within communities [40]. Greater attention is being paid to addressing implicit biases [41] and cultivating critical consciousness in medical education [42]. The ongoing emphasis on increasing transparency through standardized reporting, making datasets available, and mandatory disclosures are further indications of interest in attending to blind spots and shining light on promising pathways forward.

## Adjacent possibilities in medical education

Transforming medical education requires more than merely seeing what’s around us today with clear vision; we must also imagine with a spirit of creativity what the future will hold. Our current world could not have been imagined 100 years ago, or even 25 years ago. Likewise, we are largely in the dark to what the world will look like in the future. In Steven Johnson’s book, *Where Good Ideas Come From* [43], the author describes core principles that result in the generation of “good ideas.”

One principle is that innovative ideas occur in the realm of the “adjacent possible,” a concept first defined by theoretical biologist Stuart Kauffman [44] that refers to a future state near enough to be conceptualized but not so distant as to be unimaginable. Along the same lines, platforms for innovation that encourage connections between ideas result in the most and best new insights. Johnson summarizes Darwin’s observations of coral reefs as one such platform, wherein the more diversity and alternatives that are supported by the reef, the healthier the ecosystem is and the more likely progress is to occur. As we actively solicit diverse points of view to identify blind spots, we will inevitably create platforms for innovation, improve the medical education ecosystem, and expand our collective field of vision to bring to light more possibilities.

## How we can look for blind spots in medical education

There is no standardized way of identifying blind spots in medical education. Previous efforts to demonstrate the existence of a particular blind spot in the scholarly literature found that this could require great effort [20]. The forecasting literature offers methods of overcoming cognitive biases, such as through counterfactual thinking, systematically considering the opposite, and having an a priori framework for comparison [45]. Evidence-based strategies to reduce implicit bias include stereotype replacement, counter-stereotypical imaging, and perspective taking [46]. Suggestions for promoting critical consciousness in medical education include creating nonhierarchical environments where it is safe to question assumptions and challenge authority and using real stories as the basis for understanding how historical, geographic, and sociocultural factors can benefit some and not others [42]. Appreciative inquiry may have a role in addressing blind spots by bringing greater visibility and attention to what is being done well [47].

It will be easier to address some blind spots than others depending on the problem, context, and people involved. Those who already value evidence-based practice may welcome a greater awareness of the theories, principles, or research that are relevant to their educational work. Changes to policies that govern academic or healthcare institutions may be amenable to rational and fact-based dialogue when they align with its stakeholders’ shared goals. Blind spots that relate to one’s privileged status could be much more difficult to address, and attempting to point out such blind spots could lead to uncomfortable situations. Nonetheless, failing to attempt to see such blind spots would only perpetuate the problems that they cause.

While there are many options for identifying and addressing blind spots, the unifying principle is to ask others for help in seeing them. Asking for help is an act of vulnerability, humility, and bravery. It entails risk: a request may be ignored or the response may be unexpected, unfavorable, or unflattering. However, striving to see the world through the eyes of others creates opportunities to stop making the same mistakes and to advance. When each of us acknowledges that we are prone to ignoring our own blind spots and adopts a practice of valuing the perspectives of others, we may develop a culture in which seeking out and scrutinizing blind spots is routine. Along this path, it is imperative that we examine our own blind spots, and carefully listen, especially when what we hear creates dissonance or forces us to question strongly held beliefs. Those with status, power, and authority have the opportunity to model and lead by inviting others to challenge them and point out their blind spots.

It is impossible for any individual to know the perspectives of everyone else, but every individual can do more. We can begin by inviting feedback or new ideas from one more person involved in our daily practice than we have before, including learners, healthcare team members, patients, and caregivers. Prioritizing diversity—asking someone who might not normally be asked or whose experiences may be very different from our own—would likely lead to the most valuable insights. Structurally, we must make it easier for more stakeholders within medical education to achieve meaningful participation, not simply representation, on a regular basis. We must empower those lower in medicine’s traditional hierarchies, especially learners and patients, and those who may be disadvantaged by society’s structures, to weigh in on the future directions that medical education should take. While medical education is interdisciplinary, it is also still very insular [48]. Bringing together those from outside medical education in ways that are meaningful, lasting, and constructive could not only mitigate biases, but help us generate more creativity [49], see more “adjacent possibilities,” and select avenues to pursue innovation.

Platforms for innovation within and across institutions can also be developed to generate “good ideas.” Venues that foster spontaneous interactions and creative collisions must be valued, even when they may seem to be at odds with outcomes-oriented efficiency. Interactions can be facilitated synchronously, such as through “unconferences,” [50] in which a group first gathers around a shared interest, then collectively creates and proceeds through an agenda. These can also be conducted asynchronously, such as through social media or crowdsourcing platforms.

While normalizing the search for, and recognition of, blind spots may lead to improvement, not all blind spots can or should receive equal priority. As more of medical education’s stakeholders participate in identifying blind spots, they can also develop a core set of principles that can inform the prioritization of blind spots. Such a process may encourage those with influence or power to align incentives and resource allocation with these priorities. Increasing the breadth and diversity of perspectives involved in medical education reform may disrupt the status quo and guide us toward realizing the potential of medical education to serve society’s needs.

## Conclusions

Medical education continues to struggle to prepare a physician workforce that meets societal needs while adapting to emerging evidence and evolving clinical and social contexts. Calls for reform from groups of experts have failed to translate into the innovations that are necessary. Proactively identifying blind spots in medical education is an approach that is democratic, inclusive of all perspectives, embraces complexity, seeks transparency, and invites humility. Taking this approach may allow us to visualize more adjacent possibilities and identify the paths we need to take to advance medical education.
